# Implications of the Theory of Basic Human Values for the Second Demographic Transition: Interdependence and Individualism in the Era of Self-Fulfillment

**DOI:** 10.1007/s10680-023-09677-0

**Published:** 2023-09-01

**Authors:** Oscar Smallenbroek

**Affiliations:** European University Institute, Via dei Roccettini, 9, 50014 San Domenico di Fiesole, Italy

**Keywords:** Second demographic transition, Personal values, First marriage, Theory, Cohabitation

## Abstract

I examine the implications of a modern psychological theory of values for the Second Demographic Transition (SDT). The SDT derives its values theory and measurement from Maslow, who noted that resource-rich environments cause value shifts towards personal-focused growth values. However, Maslow has been replaced by the theory of basic human values (TBHV) which distinguishes person and social-focused growth values. This distinction has two important implications for the SDT. First, some individualistic and self-expressive values identified by the SDT are not growth but basic need motivated and therefore functions of resource-poor environments. Second, the TBHV values on interdependence and independence are strongly influenced by gender and reflect preferences for family and care or career. Therefore, these values can be used to address critiques of the SDT based on the stalled gender revolution. I show that distinguishing values as described in the TBHV can be useful for the SDT. I find that benevolence (interdependence) is positively and openness to change (autonomy/stimulation) is negatively related to marriage in the Netherlands using longitudinal panel data and discrete event history models.

The Second Demographic Transition narrative (SDT) is unique in applying an ideational lens to explain demographic changes in Europe and globally. The SDT narrative argues that an increase in self-expressive over traditional values triggered a proliferation of living arrangements, subreplacement fertility, and rising divorce rates within the economic and cultural context of the late twentieth century (Esteve et al., 2012; Lesthaeghe, 2010; Lesthaeghe & Van de Kaa, 1986; Van de Kaa, 1987).

The SDT analyses demographic behaviour at the societal and individual levels. At the societal level, the SDT integrates important insights from sociology and political science to explain demographic behaviour. The SDT documents a shift in societal norms facilitating social solidarity and gender inequality through sex-segregated roles to a society with high social disengagement and radical changes in the roles and options available to women (education, labour market, contraceptives). Equally important in the SDT are cultural changes in the meanings attributed to marriage and fertility. Before the SDT, marriage and childrearing were major life goals. Present day marriage is still a significant milestone, but mostly serves as a means for personal development (Cherlin, 2004; Giddens, 1992; Keizer & Hiekel, 2015).

The SDT relies, at the individual level of analysis, on Maslow (1943, 1999), who argued that values are a function of human needs and therefore respond to resources available in the environment and are organised hierarchically. He argued that rising material welfare generates changes in values at the individual level. Accordingly, social cohesion and tradition are replaced by individualistic, self-expressive, and nonconformist orientations. However, significant theoretical developments in psychology have replaced Maslow’s ideas.

This article is intended as a psychological approach to the individual level of analysis of the SDT. It aims to shed light on two important implications for the value dimension of the SDT by applying to it, for the first time, the theory of basic human values (TBHV) (Schwartz, 1992). Applying the TBHV to the individual-level analysis of the SDT changes our understanding of the relationship between values and the environment. The TBHV stresses that individualistic values are related to *basic needs* rather than growth needs, and therefore, occur in environments where something is lacking. The second implication is to stress a greater sensitivity to gender differences in values and the expression of those values. Using the TBHV, psychologists found large gender differences in value orientations which capture preferences for investing in a career or family formation (Borg, 2019; Milfont et al., 2016; Schwartz & Rubel, 2005). Managing the trade-off between career and family preferences is a major challenge to decrease gender inequalities in paid and unpaid labour, which in turn impact demographic behaviour (Blossfeld & Hakim, 1997; Cech, 2013). Therefore, further distinguishing value orientations in the SDT can improve our understanding of the gender dynamics of demographic behaviour (Bernhardt, 2004; Goldscheider et al., 2015; Zaidi & Morgan, 2017).

This article empirically examines the utility of the four value dimensions specified in TBHV for the SDT. It uses the Longitudinal Internet studies for the Social Sciences (LISS), a nationally representative panel of the Netherlands, to fit discrete event history models with cohabitation and first marriage as outcomes. This paper is one of few to examine the relationship between values and demographic behaviour using longitudinal data (Barber, 2001; Thornton et al., 2007) providing strong evidence for an ideational lens to understanding demographic behaviour.

In the following section, I will first outline the theory of basic human values. I then draw parallels and highlight differences in the value concepts found in the SDT and TBHV. Then I explain how the SDT can benefit from the value conceptualisation in the TBHV to further analyse the role of gender in demographic behaviour. A short review of the literature precedes the method and results section.

## The Theory of Basic Human Values

In the wake of Maslow’s contributions, three major findings profoundly reshaped the conceptualisation of values in psychology. First, Rokeach (1973) categorised values by their goal content. Second, Schwartz and Bilsky (1987) found that values can be categorised by their motivating needs. The third and most important contribution is the circumplex structure of values (Schwartz & Bilsky, 1987), which is a direct consequence of the underlying motivations and goal-focus of the values.

**Fig. 1:**
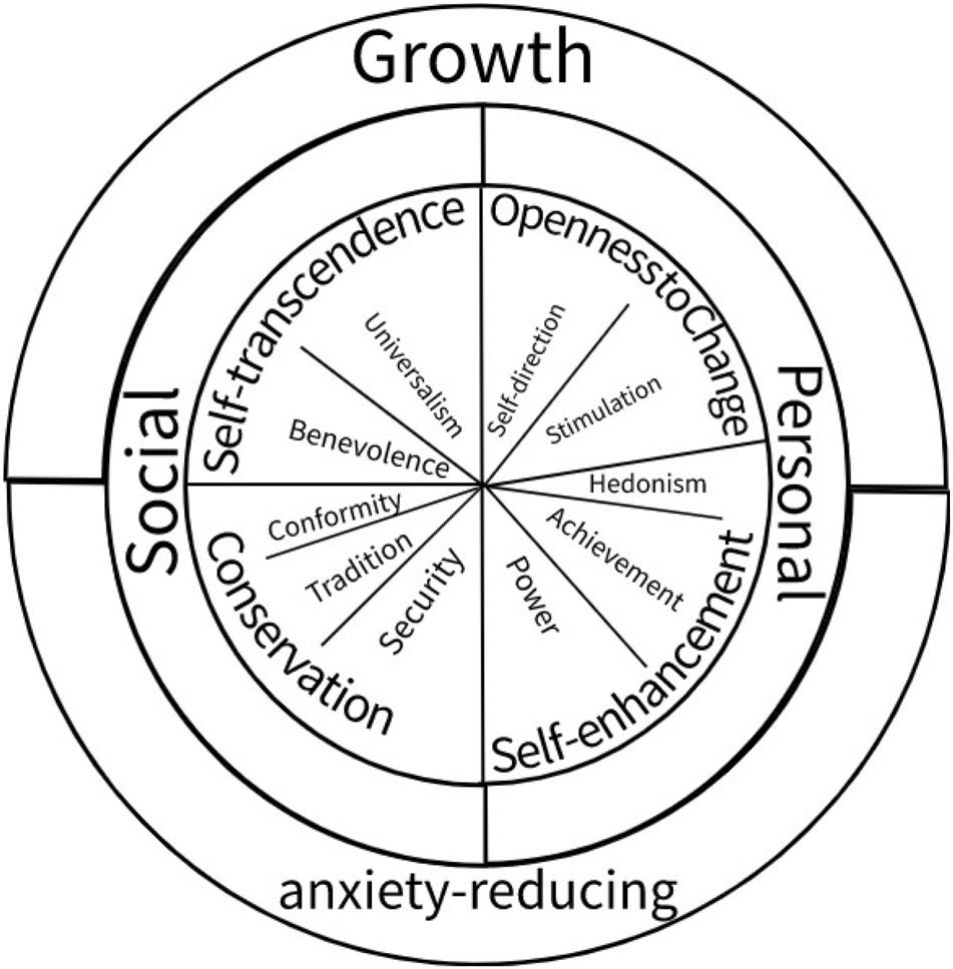
The circumplex of values in the theory of basic human values

In the circumplex structure, adjacent values are strongly related, while values separated by 180 degrees are mutually exclusive in their goals and needs. For example, the conformity and self-direction values are incompatible (see Fig. 1). Furthermore, since all values are by definition important, the circumplex structure implies that the relative importance of a value guides the actions of individuals. For example, we value social conformity even when our need for it is satisfied. However, the attainment of social conformity reduces its relative importance within the value system, making it a less salient guide to behaviour. The circular structure implies that reducing the salience of social conformity will also increase the salience of self-direction values. Consequently, Schwartz and Bilsky (1987, p. 551) defined values as ‘(a) Concepts or beliefs, (b) about desirable end states or behaviours, (c) that transcend specific situations, (d) guide the selection or evaluation of behaviour and events, and (e) are ordered by relative importance’. Schwartz (1992) has identified a set of 10 value types that can be aggregated into four higher-order orientations by combining their underlying needs (growth/anxiety-reducing) and goal focus (social/personal).

Figure 1 shows the circumplex of values. In the outer ring are the motivational need basis of values, the second ring contains the goal focus, and the third ring consists of the higher-order value orientations: openness to change, conservation, self-transcendence, and self-enhancement. Lastly, the values themselves are inside the circle. The value definitions can be found in Table 1.

**Table 1:** Value definitions and example items from the Rokeach Value Survey and Schwarz’s Portrait Values Questionnaire

	Value definition^a^	Rokeach Value Survey items used	Schwartz example Items^b^
Self-transcendence	*Benevolence*: preservation, and enhancement of the welfare of people with whom one is in frequent personal contact	helpful, loving, sincere and truthful	It’s very important to him^c^ to help the people around him. He wants to care for other people
	*Universalism*: understanding, appreciation, tolerance and protection for the welfare of all people and nature		He thinks it is important that every person in the world be treated equally. He wants justice for everybody, even for people he doesn’t know
Openness to change	*Stimulation*: Excitement, novelty, and challenge in life	open-minded, pleasure, and freedom	He thinks it is important to do lots of different things in life. He always looks for new things to try
	*Self-direction*: Independent thought and action-choosing, creating, exploring		He thinks it’s important to be interested in things. He likes to be curious and try to understand all sorts of things
	*Hedonism*: Pleasure and sensuous gratification for oneself		Having a good time is important to him
Self-enhancement	*Power*: social status and prestige, control or dominance over people and resources	a sense of accomplishment, social recognition, an exciting life	It is important to him to be rich. He wants to have a lot of money and expensive things
	*Achievement*: personal success through demonstrating competence according to social standards		It is very important to him to show his abilities. He wants people to admire what he does
Conservation	*Conformity*: restraint on actions, inclinations, and impulses likely to upset or harm others and violate social expectations or norms	clean, obedient, self-controlled	It is very important to him that his country be safe from threats from within and without. He is concerned that social order be protected
	*Tradition*: respect, commitment and acceptance of the customs and ideas that traditional culture or religion provides		He thinks it’s important not to ask for more than what you have. He believes that people should be satisfied with what they have
	*Security*: safety, harmony, and stability of society, of relationships and self		It is important to him to live in secure surroundings. He avoids anything that might endanger his safety

^a^Definitions are from Schwartz et al. (2012)

^b^PVQ21 items from Schwartz (2003:311:314)

^c^personal pronouns are matched to respondent’s gender

In the top left of the value circumplex are self-transcendence values of universalism and benevolence. These orient an individual to interpersonal connection and the welfare of the community, society, and environment. These are based on the growth needs of love, belonging, and connection (Baumeister & Leary, 1995; Koltko-Rivera, 2006; Maslow, 1999; Schwartz, 1992). Bottom right are self-enhancement values, including power and achievement. These orient an individual to attaining status, socially recognised abilities, and power to make relationships superfluous. They are based on anxiety-reducing needs such as self-esteem and respect (Leary & Downs, 1995; Maslow, 1943). On the top right are the openness to change values: self-direction and stimulation. These are motivated by growth needs of self-determination (autonomy) and stimulation and have a personal goal focus (Deci & Ryan, 2000). These are incompatible with conservation values, found in the bottom left, including conformity, security, and tradition. Conservation values address anxiety-reducing (basic) needs for personal and in-group safety by providing a predictable environment through (social) control (Schwartz, 1992).

## Applying the Basic Human Values Theory to the Second Demographic Transition

The first major difference between the TBHV and SDT literature concerns the concept of values. Whereas the TBHV excludes attitudes (criteria b), the SDT considers values and attitudes as synonymous—this widespread practise in sociology and political science can be attributed to the difficulty of measuring and linking values to behaviour (Hitlin & Piliavin, 2004; Miles, 2015). As a result, empirical work using the SDT framework often uses attitudinal items when measuring values. However, it is important to keep attitudes and values separated as this allows scholars to distinguish between cultural narratives, which provide a rationale for associating values to behaviour, and individual-level values as psychological properties of individuals.

According to Lizardo et al. (2016) and Vaisey (2009), individual values and cultural values are two separate phenomena. Individual-level values are cognitive schemas or mental representations of general ideas (Hanel et al., 2017)*.* At the cultural level, values are intersubjective ideas that are used to express individual-level values. An important implication is that the empirical relationships between values, attitudes, and behaviours depend on the cultural symbolisation of values (Miles, 2015).

The second major difference between the TBHV and the SDT is related to the value orientations. The SDT uses Maslow’s theory which does not distinguish the goal content of values from the underlying motivations. According to Maslow, personal-focused values are synonymous with growth motivations. The SDT has understandably used this conceptualisation. However, as noted earlier, the TBHV separates values along two dimensions, so that growth and basic-need motivated values can have a personal or a social- goal focus. As a result, the SDT includes a much wider array of topics within one dimension than is common in psychology.

There are clear similarities between the SDT conception of conformist versus self-expressive values and the TBHV conservation versus openness to change orientations. Both theories include, on the one hand, conformity to social norms and tradition, and, on the other hand, the pursuit of self-expression through self-directed action and stimulation seeking. However, the SDT operationalisation also includes items that belong to the self-transcendence and self-enhancement dimension within the TBHV. For instance, Moors (2008) uses items on socioeconomic success to measure autonomy and self-development values. In the TBHV. these would be categorised as power and achievement. Surkyn and Lesthaeghe (2004) code the importance of companionship in marriage and the social status of a job as conformist and self-expressive, respectively. Again, the TBHV would code these in the self-transcendence and self-enhancement orientations, respectively.

The main implication of the TBHV from the standpoint of the SDT is that some items previously labelled as individualism^1^ and self-expression and thus associated with increases in living standards are, in fact, self-enhancement or self-transcendence values. These values, moreover, signal a failure of society to meet belonging, love, and esteem needs. Thus, according to the TBHV, items, such as socioeconomic success and job prestige, are values that arise in resource-poor conditions. Therefore, it would be unwise to aggregate openness to change or self-transcendence with self-enhancement values, as the former arise in resource-rich environments.

These distinctions are not only theoretical. In fact, empirical evidence shows that self-enhancement values are related to a deficiency in needs such as deteriorating living standards (economic) or quality of life (subjective). In times of crisis, self-enhancement values tend to increase while self-transcendence values tend to decrease (Daniel et al., 2021; Park et al., 2017). Furthermore, the association between crises and self-enhancement values is mediated by welfare expenditures, being higher in low-expenditure countries (Sortheix et al., 2019). There is also evidence that value profiles, emphasising self-transcendence and openness to change, are positively associated with the human development index, health expenditure, per capita GNI, life expectancy, and government spending on education. In contrast, value profiles emphasising self-enhancement and openness to change are negatively related to the same indicators (Magun et al., 2015, 2017). In short, there is clear evidence for the utility of distinguishing the two value dimensions.

A second advantage of applying the TBHV to the SDT is that it addresses criticisms based on the stalled gender revolution (Bernhardt, 2004; Goldscheider et al., 2015; Zaidi & Morgan, 2017). Although the SDT incorporates the gender and contraceptive revolutions of the 1960s, it has paid less attention to the gendered work-family conflicts that consequently emerged. Scholars argue that the movement toward gender equality has slowed or stagnated because men do not take on care roles and tasks at a sufficient pace to compensate for changing roles of women both at home and on the job (England, 2010; Sullivan et al., 2018). The reluctance of men to assume the roles mentioned above is linked to their identities and cultural narratives on masculinity.

Personal values reflect gender-specific socialised preferences for care or career. Women generally have more social-focused values. Compared to men, women score higher on self-transcendence and conservation values while they score lower on self-enhancement and openness to change (Borg, 2019; Milfont et al., 2016; Schwartz & Rubel, 2005). These values can be used to measure an individual’s preferences for career or family formation and thus provide an ideational lens into the stalled gender revolution and its role in the SDT.

### Literature Review

To date, there are no longitudinal studies that implemented value measures that align with the TBHV and examined demographic outcomes. However, longitudinal studies generally support the arguments made in the previous section: items reflecting self-transcendence and self-enhancement values affect marriage and cohabitation. Thornton, Axinn, and Xie (2007), for example, show that men and women in the United States postpone marriage when they prefer a career over family; they also show that the effect is stronger in women. Clarkberg et al. (1995) longitudinal study of a nationally representative cohort of high school seniors in the United States found that values, such as the importance of money and leisure (self-enhancement), are negatively related to the probability of forming a first union and negatively related to marriage (instead of cohabitation)—the study started in 1972 and ran until 1986. Studies on fertility yielded similar results. Barber (2001) showed that positive attitudes toward children and childbearing increase marital childbearing, while positive attitudes toward career and luxury goods reduce rates of marital and premarital childbearing. In short, all three longitudinal studies found that items specifically related to self-transcendence and self-enhancement values are associated with marriage and childbearing.

### The Current Study and the Context of the Netherlands

Men and women in the Netherlands are relatively free to choose whether or when to cohabit and marry (Keizer & Hiekel, 2015). Cohorts born after the 1960s have predominantly formed nonmarried cohabiting unions (68%) (Feijten & Mulder, 2002; Mooyaart & Liefbroer, 2016). Despite the culturally liberal attitudes of Dutch society, childcare arrangements and taxation policies discourage women’s careers (IBO, 2019). Institutions are designed to promote semi-separate gender spheres, including the partial reimbursement of childcare, the right to parental leave and individual income taxation which encourage one and a half earner households (De Graaf & Vermeulen, 1997; Hendrickx et al., 2001). Furthermore, due to the widespread availability of part-time work and the lack of childcare options (Portegijs & Brakel, 2016) the percentage of women in full-time employment has been stable at 20% since the 1980s, although female labour market participation reached 75% in 2010 (De Graaf & Vermeulen, 1997; Khoudja & Fleischmann, 2018).

### Hypothesis

I hypothesise that benevolence (a self-transcendence value) is positively related to the probability of marriage (H1). The goal content of this value is to find stable and intimate close relationships. Achievement (a self-enhancement value) is likely negatively related to the probability of marriage (H2). The goal content of this value is to attain social standing through culturally prescribed methods, e.g., labour market success. Additionally, I expect gender differences in these effects. Men value benevolence less on average, and in the Netherlands, they are often the primary income providers. The effect of benevolence values on the probability of marriage is likely stronger for men than for women (H3). On the other hand, women face institutional barriers and considerable costs when pursuing a career. Therefore, the effect of achievement values is likely stronger for women than for men (H4).

The SDT narrative proposes that individuals choose between autonomy and social conformity when deciding to cohabit and marry. Marriage is a ‘traditional’ goal, while cohabitation before marriage breaks with tradition. Openness to change is likely to be negatively related to marriage but positively related to cohabitation (H5), while conservation is likely positively related to marriage and negatively related to cohabitation (H6).

## Data and Methods

The Longitudinal Internet Studies for the Social sciences (LISS) are carried out by the CentERdata institute and is based on a true probability sample of households drawn from the population register. Respondents participate in monthly internet surveys (Scherpenzeel & Das, 2010). Households that could not otherwise participate are provided with a computer and internet connection.

The sample for the first marriage and cohabitation analysis is respondents who are 18 to 40 years old and have never been married. Migrants of the first and second generation are excluded from the analysis. I use event history analysis to model the hazard rates of entry into cohabitation and marriage. Monthly questionnaires provide information on the ‘failure’ times of marriage and co-habitation. The data cover 2008 to 2019. Sample sizes are found in Table 2 below. Cases were dropped because the value questionnaire was not fielded in 2010, 2012, 2015, and 2018. Marriage before cohabitation was too rare (44 events) for accurate estimation of coefficients. The descriptive statistics are in the Appendix.

**Table 2:** Data selection, number of events and sample size for the any marriage and cohabitation outcomes

	Marriage		Cohabitation	
	Data	Sample	Data	Sample
Respondents (% female)	2304	2266 (56%)	1602	1563 (54%)
Observations	8720	6008	5554	3843
Events	357	261	350	238

### Variables

The LISS panel uses the Rokeach Value Survey (RVS) to measure respondents’ values (Rokeach, 1973). Respondents are asked, “Which values act as a guiding principle in your life, and which values are less important to you?” Subsequently, they are asked to rate each value on a scale of 1 “extremely unimportant” to 7 “extremely important”. There are 34 items in the questionnaire. As customary in the values literature (Schwartz, 1992; Schwartz et al., 2012), a subset of items was chosen by their location on a multidimensional scaling projection (Borg et al., 2018) in R (Mair et al., 2019), which confirmed the circumplex value structure. Each value measurement, using the items shown in Table 1, shows measurement invariance over time in a multi-group confirmatory factor analysis. The predicted factor scores are used in the analysis. Validation of the measures can be found in the supplementary material (SM).

The marriage and cohabitation events and failure times are coded such that personal values are measured before the event. The cohabitation event is defined as individuals who are the household head or partner of the household head and indicate that the household head is living with their partner. Additionally, only cohabitations of more than one year are considered.

Control variables include age, age squared, year of birth, presence of children in the household (yes/no), in-education indicator, educational attainment, national unemployment rate, employment relationship, and occupational group. These are included as time-varying covariates. Respondents who indicate that their main occupation is studying are coded as 1 in the in-education variable and otherwise 0. Educational attainment is recoded into a three-category variable: primary and lower secondary (vmbo), secondary (havo, vwo, mbo) and tertiary (hbo and wo).^2^ I also control for the quarterly seasonally adjusted unemployment rate of individuals aged 25–40, taken from the Central Bureau for Statistics Netherlands (CBS, 2020).

The current employment relationship is coded as permanent, nonpermanent (on-call and temporary), self-employed or employer, and not employed (students, unemployed, work disability). Among the unemployed, 78% are in education. Most nonpermanent contracts (80%) were temporary contracts of 29 h per week, on average.

Occupation was measured by asking respondents to assess their current or last job,^3^ which are coded into four categories: professional and managerial, white-collar, manual occupations and missing. Respondents with missing occupation data (30%) are placed in the missing category, 41% are students and 45% indicate they have a permanent contract.

### Methods

Providing evidence that personal values affect behaviour is challenging for two reasons. The first challenge relates to reverse causality: behaviour may change values. The second challenge relates to the multitude of contextual and confounding variables. I first discuss how the data and design avoid and guard against reverse causality. Then I discuss the confounding and contextual factors, and lastly the use of discrete event history models and model selection.

One way to reduce reverse causality bias is to measure the independent variables before the dependent variables. The value measures are taken between 0 and 18 months before the marriage or cohabitation event.^4^ However, measuring values before the event may not be sufficient. Marriage is often a long process that may change personal values. Therefore, I also ran models using lagged scores (see SM). In these models, values have the predicted positive or negative relationship with cohabitation and marriage. However, there are differences from the models presented in this paper. The interaction between benevolence and gender is reversed compared to the nonlagged models and most coefficients are not significant, possibly due to the reduced sample size. However, the consistency of the effects provides evidence against reverse causality.

The relative stability of values also guards against reverse causality. Personal values change slowly over time due to experience and only temporarily change in response to exogenous shocks such as an economic depression or migration (Bardi & Goodwin, 2011; Bardi et al., 2014; Lönnqvist et al., 2018; Milfont et al., 2016). The stability of personal values is apparent from correlations between measurement occasions. A longitudinal study from New Zealand found high rank-order stability (*r* = 0.60) over three years (Milfont et al., 2016). Similarly, correlations between value measurements in the LISS data range from 0.49 to 0.62 for achievement and conservation values. Benevolence and openness to change fluctuate more, the correlations between 2008 and 2019 range from 0.30 to 0.42. These correlations are likely lower than those reported by Milfont et al. (2016) due to attrition, resampling and the longer period of the LISS panel (ten versus three years).

As noted, confounding variables represent the second challenge of estimating the effects of values on behaviour. Social class and social origins are important confounding variables. Education, occupation, and social class affect openness to change and conservation values (Kohn, 1989) and the timing and probability of marriage (Liefbroer & Corijn, 1999; Mooyaart & Liefbroer, 2016). Additionally, contextual effects, such as the state of the economy, may affect values (Sortheix et al., 2019). Therefore, I control for respondents’ education, occupation, and the macro-level unemployment rate. However, social origin cannot be controlled because of a lack of data. Parents’ education and occupation were asked only in 2012 and 2013. Nevertheless, models including the father’s occupation show similar effects as those presented here. The results presented can only be interpreted as establishing empirical associations and are in no way causal relationships.

As noted, I use event history analysis to model the relationship between personal values and cohabitation and marriage. EHA models estimate the effects of covariates on the hazard rate, the rate at which an event occurs. I estimate discrete-time event history models using the complementary log–log link and cluster robust standard errors (at the person level). One set of models uses marriage as the outcome regardless of previous cohabitation. Respondents are at risk of marriage from the year they turn 18 and have never been married. Respondents exit the risk pool once married or when they turn 40. The model on marriage does not control for cohabitation status, as it may act as a collider variable (Elwert & Winship, 2014). The second model uses unmarried cohabitation as the outcome. Respondents are at risk when they indicate that they do not live with a partner, are 18 years old, and have never been married. Respondents exit the risk pool once cohabiting or when they turn 40. The age of 40 is selected as the end of the risk period as the trade-off between career and family becomes less salient. At age 35, career trajectories start to level out (Wolbers et al., 2011), while it is also the beginning of women’s fertility decline.

**Fig. 2:**
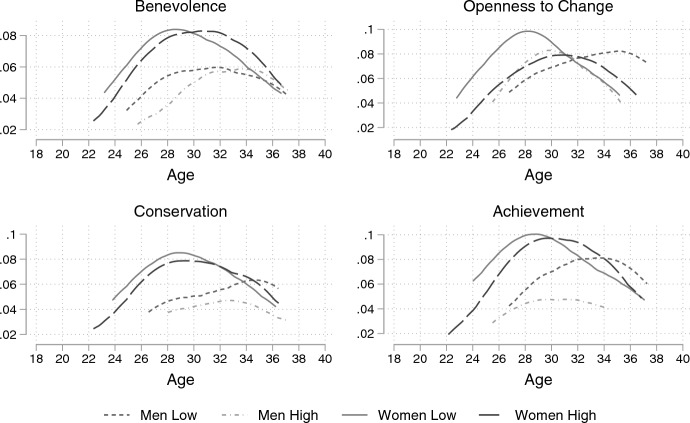
Observed hazard rate of any marriage by importance of value and gender. *Note*: Observed Nelson-Aalen hazard functions estimated by gender and high/low importance of the four values using the sample for any marriage

**Fig. 3:**
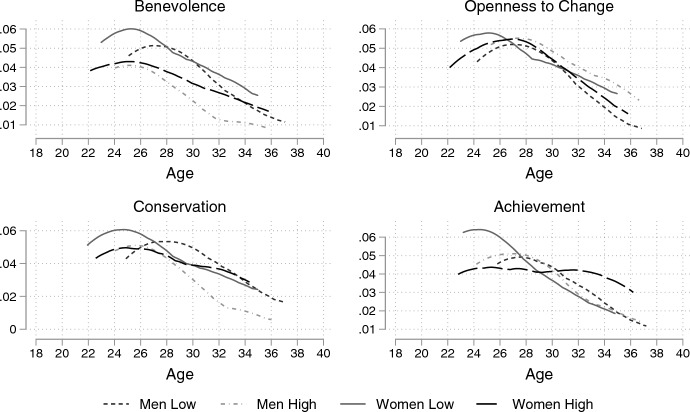
Observed hazard rate of cohabitation by importance of value and gender. *Note*: Observed Nelson-Aalen hazard functions estimated by gender and high/low importance of the four values using the sample for cohabitation

Discrete-time event history models are sensitive to the specification of the baseline hazard. The baseline hazard is not constant for the marriage or cohabitation models (see Figs. 2 and 3) but takes a parabolic form. A continuous variable (age) representing the risk time under observation and its square are included to model the baseline hazard. An interaction between age and gender is included as it is well known that women marry at a younger age than men (Billari & Liefbroer, 2010). The models were chosen on the basis of AIC and BIC. These show the observed time at risk, its square, and the interaction with gender improves model fit. To select the best model, each value measure was also interacted with time and with gender. These interaction terms did not improve the model fit. The models are robust to multicollinearity and outperform other specifications including measures using the SDT distinction between personal—social focus and using the two Schwartz value dimensions (see SM).

## Descriptive Results

In this section, I present the observed hazard curves of marriage and cohabitation. Separate curves are plotted by gender and the importance attributed to a personal value. Respondents were categorised into terciles within each wave. The highest and lowest ranking terciles are shown in the figures.

Figure 2 displays the hazard rate of marriage by the importance given to each value and gender. There are noticeable differences in hazard rates between levels of importance, and these vary by gender. As expected, the hazard rate for men who value achievement is lower than that of their counterparts. Against expectations, men who attribute high importance to benevolence initially have a lower hazard than men who value benevolence less. The ranking reverses at age 33. Women’s hazard rate is the same regardless of the importance attributed to benevolence, but the hazard rate decreases less past age 30 when benevolence is important. These patterns support the hypothesis that individuals who value relationships, and find labour market success less important, focus their efforts on building relationships. However, the timing of the peaks suggests that those who value relationships delay partnership.

The associations between marriage and conservation and openness to change values provide some support for the SDT narrative. As expected, the hazard rate of marriage is higher for women who do not value openness to change. However, there is no difference in the hazard rate of women with high or low conservation values. The association between values and men’s hazard rate of marriage does not support the SDT narrative. The hazard rate is lower for men who value conservation and does not differ between men who value openness to change compared to those who do not.

Figure 3 shows the hazard rate of entry into cohabitation by the importance of the four values and gender. Overall, the associations show an interaction between gender and values. For example, men who attribute high importance to benevolence have a substantially lower hazard rate of cohabitation, while women’s hazard rates are similar. There are minor differences in hazard rates between men, while women who value achievement less have a higher hazard rate of cohabitation.

The interaction between gender and values on the hazard rate of cohabitation is also apparent in the openness to change and conservation values. Women who value openness to change have a higher hazard rate than those who do not; however, the opposite is true for men. The hazard rate of women is identical between the high and low conservation groups, while men who value conservation have a higher hazard rate and peak earlier than men who do not value conservation. Only the association between men’s openness to change values and cohabitation supports the SDT narrative.

### Regression results

**Fig. 4:**
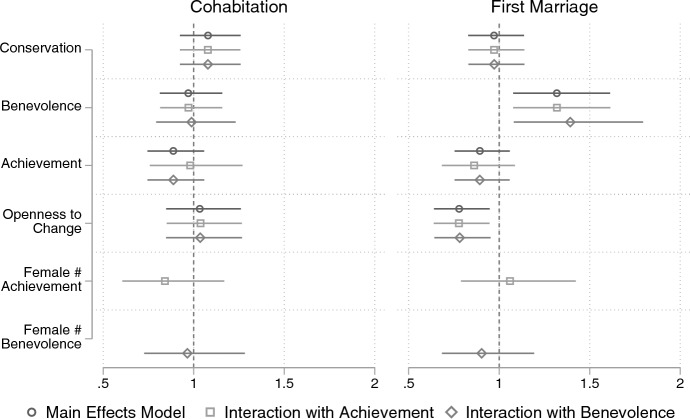
Hazard ratio of value orientations on first marriage and cohabitation from discrete event history models. *Note*: Estimated hazard ratio of cohabitation (left) and any marriage (right) from discrete event history models using a complementary log–log link and cluster robust standard errors. The models include personal value measures and control variables (main effects model), the other two models each include one interaction between a value and gender

Figure 4 shows the hazard ratios from discrete event history models (see Tables 4 and 5 in the appendix). A hazard ratio greater than 1 indicates an increase in the hazard rate with a one standard deviation increase in the independent variable. As expected, a one standard deviation increase in benevolence increases the hazard rate of marriage by 31% (H1), but benevolence does not affect the hazard rate of cohabitation. Achievement values have a small negative effect on marriage and cohabitation as expected (H2), but these effects are estimated with considerable uncertainty.

Turning to the gender differences in the effects of benevolence and achievement, we expected women’s benevolence values to have a smaller positive effect on marriage than men’s (H3) and women’s achievement values to have a greater negative effect on marriage compared to men’s (H4). As shown in Fig. 4, there are small differences in the effects of achievement and benevolence between genders, but these are estimated with such large confidence intervals that we cannot draw definitive conclusions.

Next, I consider the predictions made by the SDT. Against expectations, a one standard deviation change in conservation has a negligible effect on marriage and cohabitation. Additionally, a negligible effect of openness to change on cohabitation is estimated. The effects are estimated with considerable uncertainty and may indicate a null effect. However, as predicted by the SDT narrative, openness to change has a substantive negative effect on the hazard rate of marriage. A standard deviation increase in openness to change is associated with a 22% decrease in the hazard rate of marriage. Therefore, the original predictions of the SDT are partially confirmed.

## Discussion

In this article, I use the theory of basic human values (TBHV) to argue that we must differentiate two value dimensions when studying demographic behaviour. Demographic choices often imply a trade-off between autonomy and conformity, either following a prescribed normative life-course or alternatives. The SDT observed a societal shift in the valuation of autonomy over social conformity, disrupting the normative sequence as individuals pursue alternative goals. The SDT also posits that individuals will pursue alternative goals depending on their level of valuation of self-expressive and individualistic values. However, the SDT does not specify these alternative goals. The TBHV indicates that these alternative goals are self-transcendence (interdependence) and self-enhancement (independence) values. Including these values within the SDT can address its gender critiques, which note that values on relationships and labour market participation are important in times of the stalled gender revolution (Bernhardt, 2004; Goldscheider et al., 2015; Zaidi & Morgan, 2017). I argue that we can assess whether individuals autonomously pursue independence or interdependence by measuring self-enhancement and self-transcendence values.

In this article, I tested the predictions of the SDT using discrete event history models and distinguished four values following Schwartz (1992). Openness to change and conservation values represent the trade-off between social conformity and autonomy. Benevolence and achievement values capture the trade-off between interdependence and independence. As expected, the values of benevolence and openness to change are associated with marriage. Benevolence was found to have a positive effect on the hazard rate of marriage. Openness to change was found to have a negative effect on the hazard of marriage. There was no interaction between values and gender. However, the observed effects can explain gender differences in demographic behaviour, since the cumulative hazard is predicted to diverge over time due to the higher valuation of benevolence by women. The results indicate that benevolence values are relevant in the timing and probability of first marriage as well as gender differences.

In its original form, the SDT narrative explained the rise of cohabitation as the result of the rise of nonconformist attitudes. However, social norms and cultural narratives on marriage have changed to such an extent that cohabitation is now a signal of low commitment or a trial period before marriage rather than nonconformism (Keizer & Hiekel, 2015). Indeed, studies show that cohabitation followed by marriage and childbirth is the new normative standard (Feijten & Mulder, 2002; Mills, 2004). Therefore, the null effects of conservation values on first marriage after cohabitation and the null effect of conservation on cohabitation found in this paper fit recent scholarship on this issue. These results also highlight the importance of context and culture, which link behaviour to individual value orientations.

Unfortunately, there are some limitations to the LISS data, mainly the absence of life-history information, including the age of leaving the parental household, living alone and cohabitation with partners and/or dependent children. Therefore, it was not possible to account for all household positions identified by Surkyn and Lesthaeghe (2004) nor to test the SDT predictions on the importance of the sequencing and duration of demographic events. Previous works show that there are reciprocal relationships between household positions and value orientations due to selection and socialisation processes (Lesthaeghe, 2002; Thornton et al., 2007). Therefore, the effects found in this article reflect how differences in values are associated with entry into cohabitation and marriage on average. For example, it is likely that conservation and openness to change values still predict entry into marriage without a previous cohabitation period, as Studer et al. (2018) found using cross-sectional data, or that these same values predict entry into the first cohabitating union.

Applying the theory of basic human values to the SDT narrative provides avenues for future research. First, it provides a new lens to study cross-national differences in demographic behaviour, examining how institutions and cultural narratives moderate or mediate the relationship between personal values and demographic behaviours. For example, Reher (2019) argues that cultures that value individuals rather than family^5^ provide cultural and institutional contexts supportive of individual choice and facilitate family formation in the twenty-first century. Alternatively, in some countries, marriage is predominantly a signal of social status. Therefore, men and women may pursue marriage as an expression of self-enhancement values.

Second, incorporating values on interdependence and independence provides an opportunity to join the SDT with the stalled gender revolution (Goldscheider et al., 2015). Gender differences in values vary considerably between countries, but have rarely been explored. These factors may help explain cross-national variation in demographic behaviour and the timing and sequence of different elements of the SDT. For example, this article has shown that benevolence values (a self-transcendence value) impact the hazard rate of marriage for men and women. However, there is no research on cohort differences in self-transcendence and self-enhancement dimensions, whether these interact with gender or correspond to structural changes in female education and employment.

## Conclusions

In this article, I apply a psychological theory of values to the SDT. From the standpoint of the theory of basic human values, the SDT narrative categorises values by their goals, juxtaposing personal-focused with social-focused goals (Lesthaeghe, 2010; Surkyn & Lesthaeghe, 2004; Zaidi & Morgan, 2017). However, the TBHV identifies four value orientations, distinguished by combining their goal focus (person/social) and underlying needs (growth/basic). The separation may seem trivial as it leaves the original SDT predictions unchanged. Person-focused values are expected to decrease and social-focused values are expected to increase union formation and fertility. The crucial insight is that some items used in the SDT as representing growth orientations are, according to the TBHV, functions of basic needs and thus associated with resource deprivation. I argue that SDT could benefit from using the dimensions of the TBHV, namely self-transcendence (interdependence, social, growth)—self-enhancement (individualism, personal, basic) and openness to change (autonomy, personal, growth)—conservation (social conformity, social, basic) as they would provide greater leverage to analyse the relationship between the social and economic context of a cohort, its values and demographic behaviour. Additionally, the two dimensions provide the conceptual tools to investigate the role of gender in demographic trends, as there are large gender differences in self-transcendence and self-enhancement values. Lastly, the TBHV conceptualises values as individual properties (Schwartz, 1992), while culture is a rationale to link individual values to attitudes and behaviour (Lizardo et al., 2016; Vaisey, 2009). Therefore, incorporating the theory of basic human values has significant implications for understanding the relationship between societal and cultural contexts, value change, and demographic behaviour.

To test the utility of TBHV for the SDT, I constructed value measures using the theory of basic human values (Schwartz, 1992). I showed that benevolence (interdependence) is positively and openness to change (autonomy/stimulation) is negatively related to marriage in the Netherlands using longitudinal panel data and discrete event history models. These results support the inclusion and distinction of self-transcendence and self-enhancement values in the SDT.

## Data Availability

Register at https://www.lissdata.nl/ for access to the data. Stata syntax provided on request from the corresponding author.

## Appendix: Descriptive Statistics and Estimated Coefficients from Discrete Event History Models

See Tables 3, 4 and 5.

**Table 3:** Descriptive statistics of marriage and cohabitation samples

Variable	Marriage sample					Cohabitation sample				
	N	Mean	SD	Min	Max	N	Mean	SD	Min	Max
Age	6008	27.6	6.20	18	40	3843	25.6	6.01	18	40
Birth year	6008	1985	6.98	1968	2000	3843	1987	6.74	1968	2000
Achievement	6008	0.060	0.84	− 3.09	1.80	3843	0.11	0.84	− 3.09	1.80
Benevolence	6008	− 0.13	0.94	− 5.66	0.96	3843	− 0.15	0.97	− 5.61	0.96
Conservation	6008	− 0.27	0.91	− 3.38	1.48	3843	− 0.27	0.91	− 3.38	1.48
Openness to change	6008	− 0.12	0.86	− 5.79	0.97	3843	− 0.14	0.88	− 5.69	0.97
Unemployment rate	6008	4.11	1.38	2.30	6.90	3843	4.13	1.39	2.30	6.90
Gender	6008	1.56				3843	1.54			
Male (%)	2630	43.8				1765	45.9			
Female (%)	3378	56.2				2078	54.1			
Education	6008	2.24				3843	2.15			
Lower (%)	895	14.9				643	16.7			
Middle (%)	2753	45.8				1992	51.8			
Higher (%)	2360	39.3				1208	31.4			
Employment Relationship	6008	2.35				3843	2.69			
Permanent (%)	2841	47.3				1404	36.5			
Non-permanent (%)	583	9.70				356	9.26			
Self-Employed/Employer (%)	221	3.68				114	2.97			
Not Employed (%)	2363	39.3				1969	51.2			
In education	6008	0.30				3843	0.42			
No (%)	4235	70.5				2210	57.5			
Yes (%)	1773	29.5				1633	42.5			
Occupation	6008	2.69				3843	2.79			
Professional and managerial (%)	592	9.85				303	7.88			
White collar (%)	2479	41.3				1441	37.5			
Manual (%)	1117	18.6				852	22.2			
Missing (%)	1820	30.3				1247	32.5			
Children	6008	0.46				3843	0.52			
No (%)	3239	53.9				1853	48.2			
Yes (%)	2769	46.1				1990	51.8			

**Table 4:** Hazard ratios and confidence intervals estimated with discrete event history models with any marriage as outcome

	(1)	(2)	(3)
Age	1.41	1.41	1.41
	[1.20–1.66]	[1.20–1.65]	[1.20–1.66]
Age squared	0.99	0.99	0.99
	[0.98–0.99]	[0.98–0.99]	[0.98–0.99]
Education (ref. lower)			
Middle	0.95	0.96	0.95
	[0.60–1.52]	[0.60–1.52]	[0.59–1.51]
Higher	1.10	1.10	1.09
	[0.70–1.73]	[0.70–1.73]	[0.69–1.72]
Employment relationship			
Non-permanent	1.22	1.22	1.22
	[0.85–1.75]	[0.85–1.76]	[0.85–1.75]
Self-Employed/Employer	0.49	0.49	0.50
	[0.23–1.06]	[0.23–1.05]	[0.23–1.06]
Not employed	0.59	0.59	0.59
	[0.37–0.94]	[0.37–0.94]	[0.36–0.94]
Year of birth	1.00	1.00	1.00
	[1.00–1.00]	[1.00–1.00]	[1.00–1.00]
Occupation (ref. Professional/Managerial)			
White collar	0.81	0.81	0.81
	[0.56–1.17]	[0.56–1.17]	[0.56–1.17]
Manual	0.59	0.59	0.58
	[0.34–1.01]	[0.34–1.01]	[0.34–1.01]
Missing	1.02	1.02	1.02
	[0.68–1.54]	[0.68–1.54]	[0.68–1.53]
Unemployment rate	1.07	1.07	1.07
	[0.99–1.17]	[0.99–1.17]	[0.99–1.17]
In education	0.23	0.23	0.23
	[0.09–0.59]	[0.09–0.59]	[0.09–0.59]
Conservation	0.97	0.97	0.97
	[0.83–1.14]	[0.83–1.14]	[0.83–1.14]
Benevolence	1.32	1.32	1.39
	[1.08–1.61]	[1.08–1.61]	[1.08–1.79]
Achievement	0.89	0.86	0.89
	[0.75–1.06]	[0.68–1.09]	[0.75–1.06]
Openness to change	0.78	0.78	0.78
	[0.64–0.95]	[0.64–0.95]	[0.64–0.95]
Children in household	0.79	0.80	0.79
	[0.60–1.06]	[0.60–1.06]	[0.60–1.06]
Female	2.20	2.18	2.18
	[0.99–4.89]	[0.98–4.86]	[0.98–4.84]
Female x age	0.95	0.96	0.95
	[0.90–1.01]	[0.90–1.01]	[0.90–1.01]
Female x achievement		1.06	
		[0.79–1.42]	
Female x benevolence			0.90
			[0.68–1.19]
Observations	6,008	6,008	6,008
Log likelihood	− 966.8	− 966.7	− 966.6
events	261	261	261
AIC	1974	1976	1975
BIC	2108	2116	2116

**Table 5:** Hazard ratios and confidence intervals estimated with discrete event history models with cohabitation as outcome

	(1)	(2)	(3)
Age	1.33	1.34	1.33
	[1.17–1.52]	[1.17–1.52]	[1.17–1.52]
Age Squared	0.99	0.99	0.99
	[0.98–0.99]	[0.98–0.99]	[0.98–0.99]
Education (ref. lower)			
Middle	1.32	1.08	1.07
	[0.80–2.15]	[0.65–1.79]	[0.65–1.78]
Higher	1.69	1.24	1.23
	[1.02–2.79]	[0.74–2.08]	[0.73–2.07]
Employment Relationship			
Non-permanent	1.04	1.03	1.04
	[0.67–1.62]	[0.66–1.60]	[0.67–1.62]
Self-Employed/Employer	0.92	0.92	0.92
	[0.47–1.79]	[0.47–1.79]	[0.47–1.79]
Not Employed	0.80	0.79	0.80
	[0.48–1.35]	[0.47–1.34]	[0.47–1.35]
Year of birth	1.00	1.00	1.00
	[1.00–1.00]	[1.00–1.00]	[1.00–1.00]
Occupation (ref. Professional/Managerial)			
White collar	0.82	0.82	0.82
	[0.54–1.25]	[0.54–1.25]	[0.54–1.25]
Manual	0.87	0.88	0.87
	[0.51–1.49]	[0.51–1.50]	[0.51–1.49]
Missing	0.68	0.68	0.68
	[0.42–1.10]	[0.42–1.09]	[0.42–1.10]
Unemployment Rate	1.08	1.08	1.08
	[0.99–1.18]	[0.99–1.18]	[0.99–1.18]
In education	0.93	0.94	0.93
	[0.50–1.74]	[0.50–1.76]	[0.50–1.75]
Conservation	1.08	1.08	1.08
	[0.92–1.26]	[0.92–1.26]	[0.92–1.26]
Benevolence	0.97	0.97	0.99
	[0.81–1.16]	[0.81–1.16]	[0.79–1.23]
Achievement	0.89	0.98	0.89
	[0.74–1.06]	[0.76–1.27]	[0.74–1.06]
Openness to Change	1.03	1.04	1.04
Children in household	0.24	0.24	0.24
	[0.16–0.37]	[0.16–0.36]	[0.16–0.37]
Female	2.35	2.43	2.33
	[1.29–4.27]	[1.34–4.41]	[1.28–4.25]
Female x Age	0.94	0.94	0.94
	[0.90–0.99]	[0.89–0.99]	[0.90–0.99]
Female x Achievement		0.84	
		[0.61–1.17]	
Female x Benevolence			0.97
			[0.73–1.28]
Observations	3,843	3,843	3,843
Log Likelihood	− 796.3	− 795.7	− 796.3
events	238	238	238
AIC	1633	1634	1635
BIC	1758	1765	1766

## Footnotes

1 Lesthaeghe (1991) initially acknowledged that autonomy and individualism are different orientations, but subsequent studies do not make this distinction (Lesthaeghe, 2010; Moors, 2008; Surkyn & Lesthaeghe, 2004).

2 VMBO is pre-vocational education. HAVO and VWO are secondary education degrees which give access to tertiary education. MBO is a secondary vocational education. HBO is tertiary vocational education and WO is university level education.

3 Occupational groups are presented to respondents with examples: higher academic or independent profession (e.g. architect, physician, scholar, academic instructor, engineer), higher supervisory profession (e.g. manager, director, owner of large company, supervisory civil servant), intermediate academic or independent profession (e.g. teacher, artist, nurse, social worker, policy assistant), intermediate supervisory or commercial profession (e.g. head representative, department manager, shopkeeper), other mental work (e.g. administrative assistant, accountant, sales assistant, family care), skilled and supervisory manual work (e.g. car mechanic, foreman, electrician) semi-skilled manual work (e.g. driver, factory worker), unskilled and trained manual work (e.g. cleaner, packer) and agrarian profession (e.g. farm worker, independent agriculturalist). The higher occupations are coded as professional and managerial, the intermediate and other mental work is coded as white-collar, (un)skilled, and agrarian professions are coded as manual. In 2012 the CentERdata institute coded occupational responses into ISCO. Respondents self-categorize accurately into skill level but do not their work logic. For this reason, I collapse across the supervisory and academic or independent distinction made in the LISS data.

4 Changes in the month of administration cause a time gap of more than 12 months between the 2013 and 2014 survey waves, while the omission of the personality module in 2016 creates a longer time gap between the 2015 and 2017 surveys.

5 See Schwartz (2006) for a theory of cultural value orientations.
